# Deep learning tools predict variants in disordered regions with lower sensitivity

**DOI:** 10.1186/s12864-025-11534-9

**Published:** 2025-04-12

**Authors:** Federica Luppino, Swantje Lenz, Chi Fung Willis Chow, Agnes Toth-Petroczy

**Affiliations:** 1 Max Planck Institute of Molecular Cell Biology and Genetics, Pfotenhauerstrasse 108, 01307 Dresden, Germany; 2 Center for Systems Biology Dresden, Pfotenhauerstrasse 108, 01307 Dresden, Germany; 3 Cluster of Excellence Physics of Life, TU Dresden, 01062 Dresden, Germany

**Keywords:** Intrinsically disordered regions, Variant effect predictors, AlphaMissense, Benchmarking, Methionine start site

## Abstract

**Background:**

The recent AI breakthrough of AlphaFold2 has revolutionized 3D protein structural modeling, proving crucial for protein design and variant effects prediction. However, intrinsically disordered regions—known for their lack of well-defined structure and lower sequence conservation—often yield low-confidence models. The latest Variant Effect Predictor (VEP), AlphaMissense, leverages AlphaFold2 models, achieving over 90% sensitivity and specificity in predicting variant effects. However, the effectiveness of tools for variants in disordered regions, which account for 30% of the human proteome, remains unclear.

**Results:**

In this study, we found that predicting pathogenicity for variants in disordered regions is less accurate than in ordered regions, particularly for mutations at the first N-Methionine site. Investigations into the efficacy of variant effect predictors on intrinsically disordered regions (IDRs) indicated that mutations in IDRs are predicted with lower sensitivity and the gap between sensitivity and specificity is largest in disordered regions, especially for AlphaMissense and VARITY.

**Conclusions:**

The prevalence of IDRs within the human proteome, coupled with the increasing repertoire of biological functions they are known to perform, necessitated an investigation into the efficacy of state-of-the-art VEPs on such regions. This analysis revealed their consistently reduced sensitivity and differing prediction performance profile to ordered regions, indicating that new IDR-specific features and paradigms are needed to accurately classify disease mutations within those regions.

**Supplementary Information:**

The online version contains supplementary material available at 10.1186/s12864-025-11534-9.

## Background

The recent AI breakthroughs of AlphaFold2 and Generative Pretrained Transformers (GPTs) have revolutionized biomedical research. AlphaFold2 (AF2) can now generate 3D structural models for any protein of interest. Nevertheless, there are regions in proteins that do not fold into well-defined structures (i.e. disordered regions), and thus are predicted with low confidence by AF2. Disordered regions are prevalent in the protein universe, with ~ 60% of proteins in Swiss-Prot containing Intrinsically Disordered Regions (IDRs) of minimum 10 residues long [1]. In humans ~ 30% of the proteome contains disordered regions [2, 3]. Many mutations in disordered regions are associated with diseases such as breast and ovarian cancer, cardiovascular and neurodegenerative diseases, and many others [4]. An estimated 10–20% of disease mutations occur in such regions [5, 6], underscoring the importance of assessing the impact of variants in disordered regions.

Variant Effect Predictors (VEPs) are machine learning, and more recently, deep learning models that predict the pathogenicity of genetic variants. For example, they can predict the effect of missense variants (that cause an amino acid change in the protein sequence), classifying them as either pathogenic, causal for the disease phenotype or benign, not causal for the disease. The evidence of pathogenicity assessment by VEPs is included among the American College of Medical Genetics (ACMG) [7] and the Invitae semiquantitative, hierarchical evidence-based rules for locus interpretation (Sherloc) [8] clinical guidelines, and it is considered to be an in *vitro* diagnostic device (IVD) according to the European regulation 2017/746 [9].

Initially, the traditional paradigm employed by VEPs to interpret variants’ pathogenicity has been based on evolutionary sequence conservation [10, 11]. Specifically, if the protein residue is conserved, i.e. not/rarely mutated in homologous proteins in other species, it is considered important in maintaining its function. For instance, features such as the number of residues observed at the mutated position in the multiple sequence alignment (MSA) of homologous sequences are used for the interpretation of variant effects. While this traditional paradigm is still used today, it has been largely superseded by more advanced models that incorporate structure-based features.

Supervised machine learning tools for variant effect prediction (VEPs) have extended the traditional paradigm of conservation by incorporating structure-based features such as the normalized surface area of residues, protein-protein interaction sites, and secondary structure annotations [12–15]. Originally, these tools were limited by their reliance on experimental structures in structural databases, such as the Protein Data Bank (PDB), which are mainly available for conserved protein domains [12, 13]. Most recent tools took advantage of AlphaFold2 structural models and successfully improved variant effect assessment [16], especially with respect to specificity, thus minimizing the false negative rate [17, 18].

In contrast to machine learning models, which require a priori knowledge and manual curation of input features, (i.e. the regressors of the model), such as residue conservation and secondary structure assignment, deep learning models perform the extraction of features as part of the training process. More recently, deep learning VEPs were developed using evolutionary information from MSAs as input, including the protein language model-based ESM1b [19], neural network-based MVP [20], and variational autoencoder-based EVE [21]. AlphaMissense [18], the most recent deep learning VEP, combines unsupervised and supervised components of computational models. The unsupervised component leverages evolutionary information, population frequency data, and structural context derived from AlphaFold2 models, and the supervised part calibrates the output of the unsupervised part on clinical data to define a probability of pathogenicity. The recent advancements in machine and deep learning models, especially AlphaMissense, and the integration of AlphaFold2 [22] structural models have significantly improved the accuracy and specificity of variant effect prediction tools, enabling more reliable assessments of the functional impact of genetic variants.

Since AlphaFold2 models are predicted with low confidence for disordered regions, the question arises if AF2-based VEPs perform reliably on variants in disordered regions. While benchmarking of VEPs on clinical variants are abundant [10, 23–26], we found few systematic benchmarks on clinical variants in disordered regions [6, 27–29]. Such low-complexity or disordered regions pose a challenge for conservation- and structure-based VEPs, and several studies have observed decreased sensitivity of VEPs such as PolyPhen2 and SIFT when predicting mutations in intrinsically disordered regions (IDRs) [28, 29].

The aim of this study is to evaluate whether the recent AI advances, particularly that of AlphaMissense, have led to an improvement in identifying pathogenic mutations in disordered regions. To this end, we include three different computational tools for defining disorder and both machine and deep learning VEPs to evaluate their performance on benign and pathogenic mutations from ClinVar [30]. We observe, that all state-of-the-art VEPs predict pathogenic mutations in disordered regions with lower sensitivity than in ordered ones. Their overall high performance is due to over-prediction of benign variants in disordered regions, aligning with the conservation and structural paradigm that does not account for pathogenicity in less conserved and less structured regions. This analysis offers an opportunity for future development of VEPs to design models that capture protein properties not in isolation but as dynamic units of a more complex system.

## Methods

### Clinical variants

We downloaded the ClinVar VCF file (version 20231217.vcf, https://ftp.ncbi.nlm.nih.gov/pub/clinvar/vcf_GRCh37/archive_2.0/2023/clinvar_20231217.vcf.gz*)* and the variant summary file (https://ftp.ncbi.nlm.nih.gov/pub/clinvar/tab_delimited/archive/2023/variant_summary_2023-12.txt.gz*)* and we kept only variants with either a ‘pathogenic’ or ‘benign’ clinical significance (including likely benign and likely pathogenic labels). Variants with conflicting interpretations, Variants of Uncertain Significance (VUSs) and without a clinical significance label were excluded. Further, we excluded variants which had only somatic labels.

We used MapSNPs annotation tool from PolyPhen-2 v2.2.3 [12] (http://genetics.bwh.harvard.edu/pph2/dokuwiki/downloads) to map the genome assembly hg19/GRCh37 variants’ coordinates to missense coding SNPs. Only variants mapping to known canonical transcripts according to the UCSC Genome Browser were retained. We used the PolyPhen-2 v2.2.3 pipeline to annotate features (http://genetics.bwh.harvard.edu/pph2/dokuwiki/downloads). A complete list and description of the features are available at the PolyPhen-2 v2.2.3 Wiki page (http://genetics.bwh.harvard.edu/wiki/pph2/appendix_a). Only variants in the dataset with predictions from all five computational disorder tools were annotated, resulting in a final set of 61878 variants, 23234 pathogenic and 38644 benign, mapped to 7459 proteins. On average, there are 8 variants per protein. The dataset can be downloaded from the Git repository (https://gitlab.mpi-cbg.de/tothpetroczylab/idrs_veps).

### Pathogenicity scores

Pathogenicity scores were obtained using the dbNSFP47a [31, 32] command-line application, downloaded from http://database.liulab.science/dbNSFP#version. We have downloaded scores for VEST v4.0, PolyPhen-2 v2.2.3 (HVAR), REVEL, ESM1b, MVP, VARITY_R, EVE and MutPred. To calculate the accuracy, we thresholded the scores as recommended by the authors. For REVEL [33], VEST4 [34], VARITY_R [13] the cutoff is 0.5. For ESM1b [19] the threshold is -7.5 as described in their paper. For MVP a recommended score of 0.7 was used. AlphaMissense directly provides discretized predictions with three categories: benign, pathogenic and ambiguous. We excluded variants in the ambiguous category. AlphaMissense predictions were downloaded from https://console.cloud.google.com/storage/browser/_details/dm_alphamissense/AlphaMissense_aa_substitutions.tsv.gz. We considered both deep and machine learning VEPs. We defined deep learning models as models whose features extraction is automated and part of the training algorithm. Those models are AlphaMissense, MVP and ESM1b. Machine learning models, such as VEST, VARITY and PolyPhen rely on manual annotation of features. We excluded VEPs with more than 50% of missing predictions for ClinVar variants according to dbNSFP, namely EVE [21] and MutPred (http://mutpred.mutdb.org/)(Supplementary Fig. S4).

### Disorder scores

We considered five different computational predictors of disorder (AIUPred [35], AlphaFold2 [22] pLDDT scores, metapredict [36, 37], AlphaFold2-RSA [38] and flDPnn [39]. For metapredict and AIUPred, residues with disorder scores greater than 0.5 were considered disordered, while for AlphaFold2 pLDDT a score lower or equal to 70 was used to identify disordered residues. For AlphaFold2-RSA the threshold recommended by the authors is 0.581 and for flDPnn is 0.3. Based on metapredict, flDPnn and AIUPred model predictions, only 4%, 5% and 1% of disordered residues are not contained within an intrinsically disordered regions (IDRs) of at least 10 residues (Supplementary Fig. S1). Consequently, we have concluded that both disorder definitions are interchangeable and we refer to variants associated with disordered residues or to variants in disordered regions as synonyms.

For both flDPnn and AIUPred, the identification of an IDR was achieved using the Savitzky-Golay filter (moving average window size = 9, polynomial degree = 3). The disorder threshold was set at 0.3 and 0.5, respectively, and consecutive disordered residues were concatenated to form IDR segments. Disorder segments shorter than 10 residues were filtered out. IDRs were divided into four distinct groups: N-terminal, C-terminal and between domains IDRs, and intrinsically disordered proteins (IDPs). The N-terminal IDR was annotated if the protein sequence started with an IDR, the C-terminal IDR corresponded to the IDR that ended with the last residue of the protein, and IDRs located between non-disordered segments were annotated as IDRs between domains. An IDP is defined as a protein whose entire sequence is annotated as an IDR.

### Metapredict

Metapredict v2.6 was downloaded from the github repository available at the link https://github.com/idptools/metapredict/tree/master, the functions metapredict-predict-disorder and metapredict-predict-idrs were used. The output file was processed with a custom script to obtain the desired dataframe format with three columns: UniProt accession id, residue position and metapredict score. The metapredict score threshold to define disordered residues is 0.5. The function to predict IDRs was parsed with custom scripts to obtain a format with three columns: UniProt accession id, start residue of the IDR and end residue of the same. All the scripts used to run and parse metapredict files are available at the Git repository.

### AIUPred

AIUPred was run with the command line tool downloaded from here https://aiupred.elte.hu/. The scripts used to run and parse AIUpred files are available at the Git repository. We used a threshold of 0.5 to consider a residue disordered.

### AlphaFold2 pLDDT

pLDDT scores were extracted from AlphaFold2 v4 models with a custom R script. Proteins longer than 2700 residues have multiple AlphaFold2 3D models that we combined together to obtain the full-length model of the protein. Only for one protein (Q8IVF2) the full model was not successful. All the scripts used to run and parse AlphaFold2 3D models files are available at the Git repository.

#### AlphaFold-Relative solvent accessibility (RSA)

AlphaFold-RSA was run as a python script from the Git repository https://github.com/BioComputingUP/AlphaFold-disorder. Although not specified, we could only successfully run the script with the following version of dependencies, Pandas version 1.5.3, NumPy version 1.21.6, BioPython version 1.85 and Python version 3.10.16. We used the default parameter to run the tool, namely a window of 25 residues to smooth the RSA values. The disorder threshold recommended by the authors is 0.581.

#### FlDPnn

flDPnn tool was run both as a docker container following the documentation at the Git repository https://gitlab.com/sina.ghadermarzi/fldpnn_docker and with the webserver available at this link (https://biomine.cs.vcu.edu/servers/flDPnn/) for the proteins that did not successfully run with the docker. The binary prediction of disorder propensities is provided as extra column in the output file and corresponds to a cutoff of 0.3 of the disorder propensities.

### VEPs benchmarking on clinvar variants

The ClinVar benchmark was performed on all mutations except for those occurring at the N-Methionine site, which were benchmarked separately. The number of mutations occurring at the N-Methionine sites is 523, however the number of variants for which all the VEPs had a prediction was 359, 334 pathogenic and 25 benign variants. The threshold to classify a variant either as benign or pathogenic is reported in the ‘Pathogenicity scores’ section. We reported sensitivity and specificity as VEPs performance metrics (Fig. 6) as well as F1-score and ROC-AUC (Supplementary Fig. S7).

The ClinVar set consisted of 61,878 variants. However, in order to make the benchmark comparable we included only the variants for which a prediction existed across all VEPs. This led to a benchmarking set of 45,316, 17,977 pathogenic and 27,339 benign variants. Because the distribution of ClinVar variants by disorder group and phenotypic effect is unbalanced, we calculated performance metrics, namely sensitivity and specificity, on 200 bootstrap samples. Each bootstrap sample consisted of 12,540 variants, sampled with replacement in each of the four combinations formed by two binary variables: pathogenic (benign) with disorder, and pathogenic (benign) with order. The resulting bootstrap sample size of ~ 50,000 variants recapitulate the ClinVar set size.

VEPs performance metrics from the confusion matrix:

**Table:** 

		Ground-truth	
		Pathogenic	Benign
Predicted	Pathogenic	TP	FP
	Benign	FN	TN

Sensitivity/Recall = TP/(TP + FN).

Specificity = TN/(TN + FP).

Precision = TP/(TP + FP).

F1-score = 2*precision*recall/(precision + recall).

For the AUC-ROC we used the R package pROC (version 1.18.5).

The statistical tests used in the analysis are the Wilcoxon and Chi-Square tests from the built-in stats R package (version 4.4.2).

### Motif prediction of the heat-shock beta-1 protein of HSPB1 (P04792) using SHARK-capture

Multiple sequence alignment (MSA) of the heat-shock beta-1 protein of HSPB1 gene. The MSA was obtained with the EVcouplings [40] server (https://v2.evcouplings.org/*)* with default parameters and a bit score of 0.7.

Redundant sequence of P04792 MSA were removed using cd-hit (v4.8.1) with default settings (identity threshold of 90%). On these sequences, SHARK-capture (v2.0.1) was run with default settings and k-mers post-processed with extension of overlapping k-mers. K-mers mapped to the structured region were removed [41].

## Results

### Computational tools of disorder

In order to systematically characterize variants in disordered regions, we first identified those regions using different criteria using various tools (AlphaFold2 (AF2) pLDDT [22], AIUpred [35], metapredict [37], AlphaFold2-RSA [38] and flDPnn [39] see Methods). Those tools ranked among the best disorder predictor tools in the recent benchmark on computational disorder predictors, the Critical Assessment of protein Intrinsic Disorder prediction (CAID) [42]. AIUPred is based on biophysical principles of a sequence, pLDDT scores are derived from AF2 models, and metapredict is a metapredictor that combines several disorder prediction scores including AIUPred and AF2 pLDDT. AlphaFold2-RSA calculates the relative solvent accessibility (RSA) with DSSP [43] on AlphaFold2 3D models, while flDPnn relies on secondary structure prediction from PSIPRED [44], disorder scores predicted by IUPred [45], and evolutionary information calculated with PSI-BLAST [46].

**Fig. 1:**
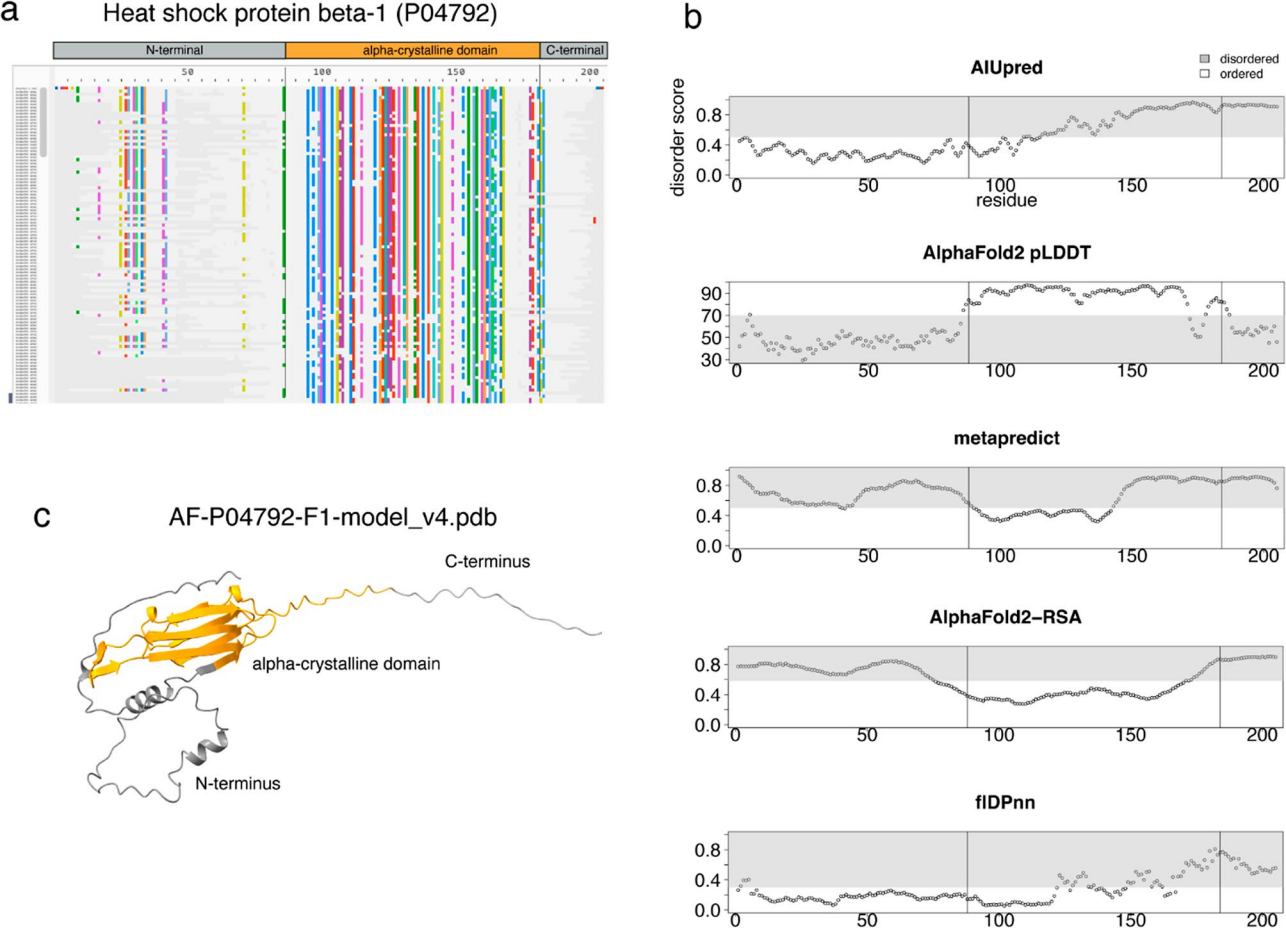
Definitions of disordered regions based on sequence conservation, computational tools and structural information largely agree. **a**) Multiple sequence alignment (MSA) of the heat-shock beta-1 protein of *HSPB1* gene. The MSA was obtained with the EVcouplings [40] server (https://v2.evcouplings.org/*)* using a bit score of 0.7. The rectangle bar over the MSA shows the domain organization according to InterPro [50] and the work of Jehle et al. [64]. The alpha-crystalline domain presents a conserved alignment, namely each homologous sequence (the rows of the MSA) has the same or similar residue as the heat-shock beta-1 protein as indicated by the colored vertical lines. Conversely, the N- and C-termini of the protein present a poor alignment, that is only few residues are conserved in the homologous sequences and the rest of them vary or are missing (shown as gaps). **b**) Disorder scores according to AIUpred, AlphaFold2 (AF2) pLDDT, metapredict, AlphaFold2-RSA and flDPnn (shown on the y-axis) for each residue position (shown on the x-axis). The grey areas highlight the disordered regions according to the specific thresholds for each tool: AIUPred and metapredict 0.5, AlphaFold2 pLDDT 70, AlphaFold2-RSA 0.581 and flDPnn 0.3. There is strong agreement on the disorder composition of the protein’s C-terminal domain, while the N-terminus is predicted to be disordered by AF2 pLDDT, metapredict and AlphaFold2-RSA but not by AIUPred and flDPnn. For instance, the alpha-crystalline domain is predicted mainly as disordered by AIUPred and flDPnn despite having a high quality x-ray crystal structure (PDB: 3Q9Q). **c**) The AlphaFold2 model of the heat-shock beta-1 protein is colored according to the domain architecture. The two termini mainly constitute disordered regions/coils (grey) while the alpha-crystalline domain (orange) is composed of beta-sheets

**Fig. 2:**
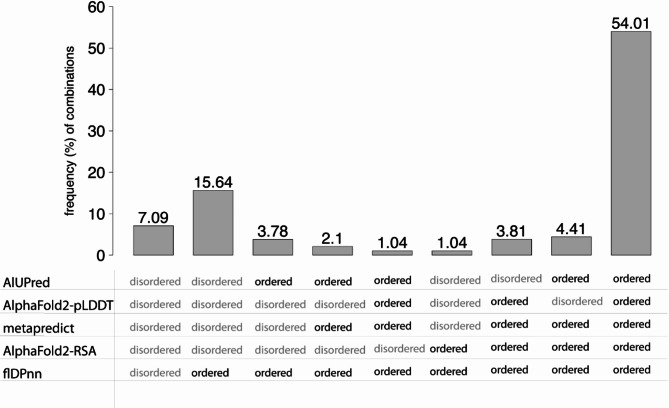
Only 7% of residues are consistently predicted as disordered across all tools. The barplot shows the frequency (%) of the combinations of disorder and order predictions among the five computational tools. Only the combinations with at least 1% frequency are shown out of the 32 possible combinations of ordered and disordered class). For example, the combination with the highest frequency (54%) is where all tools predict ordered residues

From a sequence perspective, disordered regions are characterized by lower sequence conservation, whilst from a structural point of view they exhibit mainly flexible linkers, tails (i.e. termini) and coil elements. As an example, the multiple sequence alignment of the heat-shock protein beta-1 (P04792) shows that the alpha-crystalline domain of the protein is conserved while the N- and C-termini have low-quality alignments with many gaps (Fig. 1a). The termini are associated with higher disorder scores than the domain region, according to AF2 pLDDT, metapredict and AlphaFold2-RSA but not to AIUPred and flDPnn. While this is just one example, it reflects the generally higher propensity for disorder in the N- and C-termini of proteins, which has already been assessed [47, 48]. Furthermore, this example highlights how computational disorder predictors differ in their predictions (Figs. 1b and 2 and Supplementary Fig. S2). The N-terminus of the protein is predicted as disordered by AF2 pLDDT, metapredict and AlphaFold2-RSA but not AIUPred and flDPnn, while the C-terminus is consistently identified as disordered by all tools. This is why, we use five different computational predictors to measure disorder, i.e., AIUPred [35], AF2 pLDDT [22, 49], metapredict [36, 37], AlphaFold2-RSA [38] and flDPnn [39]. The correlation between disorder tools is high overall. The lowest correlation, -37%, is observed between AF2 pLDDT and flDPnn (Supplementary Fig. S2). In general, the lowest correlations are between flDPnn and any other disorder predictor tool. The high correlation of AF2 pLDDT and AIUPred with metapredict − 76% and 77% respectively, is expected given that metapredict includes both scores during training (Supplementary Fig. S2).

The concordance among tools is principally attributable to order predictions, with 54% of residues being jointly predicted as ordered (Fig. 2). In accordance with the low correlation with flDPnn, 15% of residues are predicted as disordered by all tools except flDPnn. Additionally, all five tools label a residue as disordered in only 7% of cases (Fig. 2). The disagreement among the tools accounts for 38% of residues, which is why the inclusion of AIUPred, AF2 pLDDT, metapredict, AlphaFold2-RSA (AF2-RSA) and flDPnn in the variants benchmark is necessary to allow robust assessment and interpretation of the results.

### Variants in disordered regions are predominantly benign

**Fig. 3:**
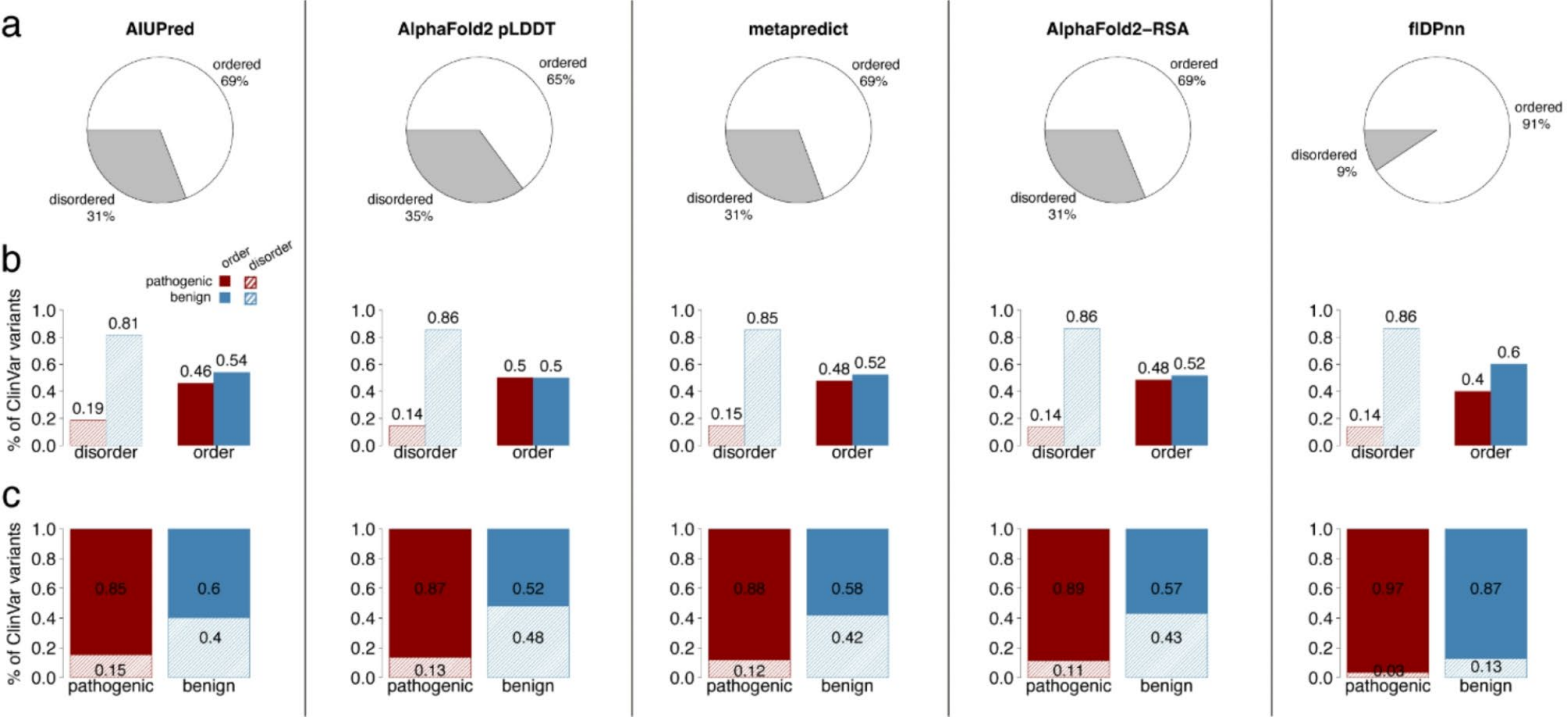
ClinVar variants that occur in disordered regions are predominantly benign. The columns correspond to the disorder definitions according to the different tools: AIUPred, AlphaFold2 pLDDT, metapredict, AlphaFold2-RSA and flDPnn. The rows show various descriptive statistical measures of the disorder and ClinVar variants. **a**) The first row shows the proportion of ClinVar variants in ordered and disordered regions, which confirms previous works reporting 30% of the human proteome as disordered [2, 3]. The exception is flDPnn, which predicts only 9% of residues as disordered. **b**) The second row shows the proportion of benign and pathogenic variants in ordered and disordered regions respectively. It highlights that more than 80% of variants in disordered regions are benign in ClinVar. **c**) The last row shows the distribution of disordered and ordered residues for pathogenic and benign variants confirming the frequently reported range of 10–15% of disease-causing mutations occurring in disordered regions [5, 6]

In order to assess the effects of missense variants in disordered regions, we collected data from a curated clinical variant database, ClinVar (see Methods). The stratification of variants showed that residues in disordered regions are abundant, with 31%, 35%, 31%, 31% and 9% of variants falling in disordered regions according to AIUPred, AF2 pLDDT, metapredict, AF2-RSA and flDPnn respectively (Fig. 3a). The variants associated with disordered residues are predominantly benign, 81% according to AIUPred and 86% for AF2 pLDDT, AF2-RSA and flDPnn, 85% for metapredict (Fig. 3b). In addition, except for flDPnn, we confirmed that 11–15% of pathogenic mutations occur in IDRs (Fig. 3c) [5, 6]. This is in agreement with finding reduced negative selection in IDRs when analyzing polymorphisms from population data of human and yeast [51].

Those observations align with the higher tolerance of IDRs to amino acid substitutions when looking at orthologous sequences of distant species. Disordered regions tend to evolve faster [52, 53] and also tolerate more insertions and deletions than ordered domains [54]. This can be attributed to the reduced evolutionary constraints on their sequences, as they are not limited by the structural requirements that constrain ordered regions. Overall, disordered regions tolerate more polymorphisms and accumulate mainly benign mutations.

### Mutations at the N-Methionine site are excessive and misclassified by VEPs

**Fig. 4:**
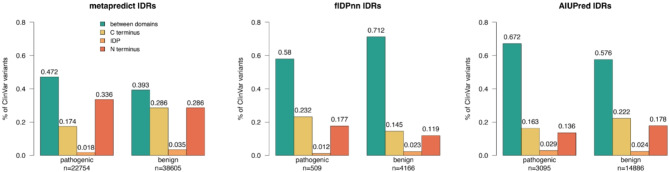
Pathogenicity enrichment in IDRs depends on the disorder prediction tool. Variants phenotypic effect (pathogenic and benign) on the x-axis according to IDR groups (C-terminal, N-terminal, between domains IDRs and Intrinsically Disordered Proteins (IDP)) and across different definitions of IDRs (metapredict, flDPnn and AIUPred). On the y-axis the marginal proportion of ClinVar variants for the corresponding category. For example, according to metapredict 34% of pathogenic variants occur in N-terminal IDRs

Since IDRs often flank structured domains as N- and C-terminal regions or connect them as linkers, we wanted to test if there is a bias in the occurrence and phenotypic effect of variants along the protein sequence, particularly at the termini. For this purpose, we classified IDRs in four groups, that is N-terminal, between domains and C-terminal IDR and IDP (Intrinsically Disordered Proteins) according to the definition given in the Methods section. We conducted a chi-square test to examine the association between protein regions (C-terminus, between domains, IDP and N-terminus) and the phenotypic effect (pathogenic and benign). The test was significant (p value < 1e-15) and the standardized residuals revealed large deviations from the expected count under the independence assumption between protein regions and variant effect. According to flDPnn and metapredict, pathogenic variants are enriched in the N-terminal IDR but depleted for AIUpred. For metapredict and AIUpred, pathogenic variants are as well more frequently found in the IDRs between domains while benign variants are more commonly located in C-terminal IDRs (Fig. 4). While for flDPnn, benign variants are more frequently observed in IDRs between domains and less in the C-terminus.

**Fig. 5:**
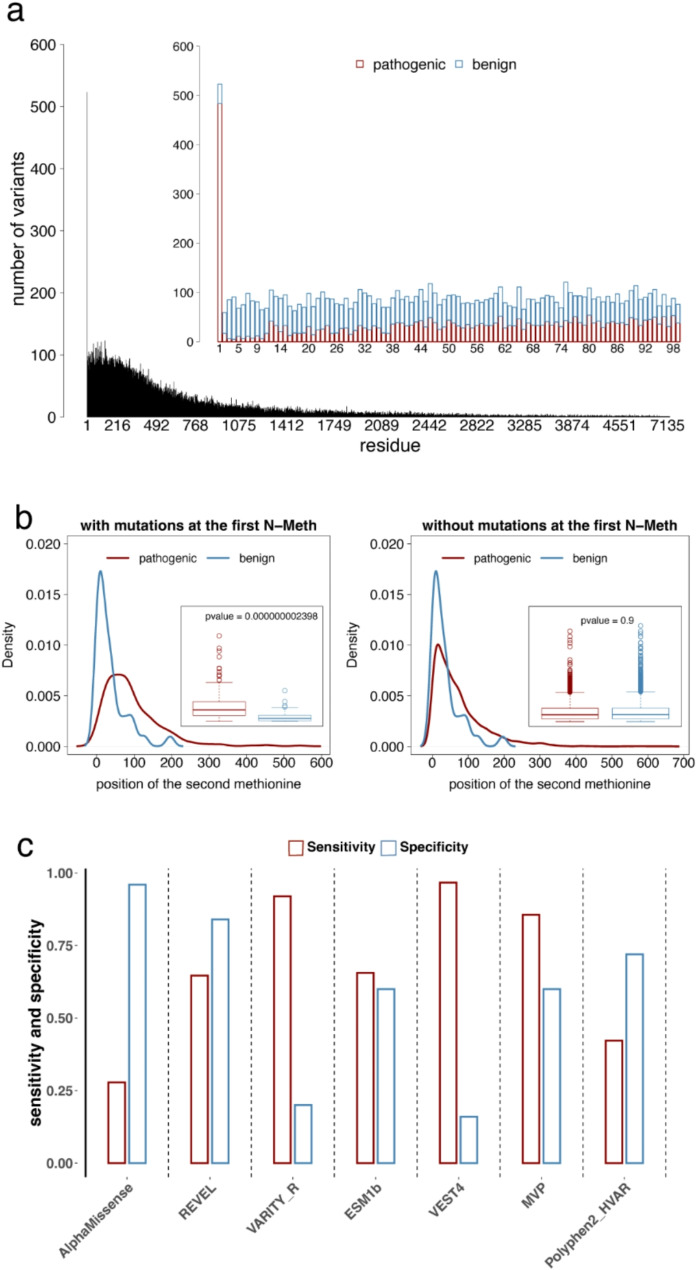
Mutations at the N-Methionine site are prevalent and misclassified by VEPs. **a**) The histogram shows the frequency of ClinVar variants (*n* = 61878) per residue position for 7459 proteins. The inset histogram highlights protein residues 1-100 and shows the number of pathogenic (red) and benign (blue) variants at each position. The prevalence of mutations at the N-Methionine site stands out with more than 500 variants associated with this site. **b**) The distribution of distances from the first Methionine to the second one for proteins with mutations at the N-Methionine site, grouped by phenotypic effect, namely pathogenic (*n* = 480) and benign (*n* = 39). In the inset, the same data is represented with a boxplot and the significance of the difference of the two distributions is annotated on the top (Wilcoxon rank sum test with one side, greater as alternative hypothesis). On the right, the same pair plots are shown for proteins without mutations at the N-Methionine site (pathogenic, *n* = 22754 and benign, *n* = 38605). The distance to the second Methionine discriminates between pathogenic and benign variants associated with the N-Methionine site and not with mutations at other sites. **c**) Sensitivity and specificity for VEPs on predicting the effect of mutations occurring at the N-Methionine site (*n* = 356 variants, including 331 pathogenic and 25 benign with prediction available across all VEPs considered in this study). AlphaMissense overpredicts benign variants and reaches only 29% sensitivity, VARITY overpredicts pathogenic variants reaching only 20% specificity, and REVEL is the most balanced with 65% sensitivity and 84% specificity

We found that the number of variants and their effect along protein sequences are not uniformly distributed (Fig. 5a). Strikingly, we observed that 0.85% of variants occur at the N-Methionine site (Fig. 5a). At first glance, this percentage may seem negligible. However, assuming a discrete uniform distribution for the event of mutations occurring in a protein of length *N* = 572, the median length of ClinVar proteins in this analysis, each site has an approximate probability of 0.17% (~ 1/600) of being mutated. In comparison, the observed frequency of 0.85% suggests a significantly higher propensity for mutations to occur at the start codon.

Variants at the starting methionine position are mainly pathogenic (93%) (Fig. 5a). Moreover, pathogenic mutations at the N-Methionine site are biased towards proteins that have a second Methionine located further away compared to proteins with benign mutations at the same site (Fig. 5b, Wilcoxon rank sum test, one-sided test with “greater” as alternative hypothesis, p-value < 2.398e-09). We performed the same analysis on proteins without mutations at the N-Methionine site, and, in this case, the distances to the second Methionine do not differ among sites with pathogenic and benign mutations (Fig. 5b). These observations suggest that a second and proximal N-terminal Methionine could compensate for the loss of the N-Methionine and perhaps serve as an alternative initiation codon [55].

Due to the unique features of the start codon, we investigated the performance of VEPs on the set of N-Methionine sites harboring pathogenic variants associated with disordered residues. Most VEPs do not perform well on mutations occurring at the N-Methionine site. In particular, we observed a strong disproportion between their sensitivity and specificity (Fig. 5c). For instance, AlphaMissense overpredicts benign variants and reaches only 29% sensitivity. VARITY overpredicts pathogenic variants reaching only 20% specificity (Fig. 5c). REVEL is the most balanced with 65% sensitivity and 84% specificity. These observations are consistent with previous work that considered variants at the first codon to be a special case of mutations, which should be analyzed with a different set of features with respect to mutations occurring anywhere else in the protein [56, 57]. For example, the position of the second Methionine in the protein sequence as well as the number of AUG codons in the 5’ UTR region of the mRNA sequence are relevant predictive features to distinguish between pathogenic and benign variants.

Accordingly, we propose that N-Methionine mutations should be treated separately as their own class of mutations because of different features that would discriminate between the pathogenic and benign variants at this position. In addition, according to metapredict and AIUPred 65% and 32% of N-Methionine sites belong to an N-terminal IDR (Supplementary Fig. S3). Since the disordered nature of the first residue might bias our investigation on VEPs, we excluded all N-Methionine mutations from all subsequent analyses.

### The gap between sensitivity and specificity is highest in disordered regions

**Fig. 6:**
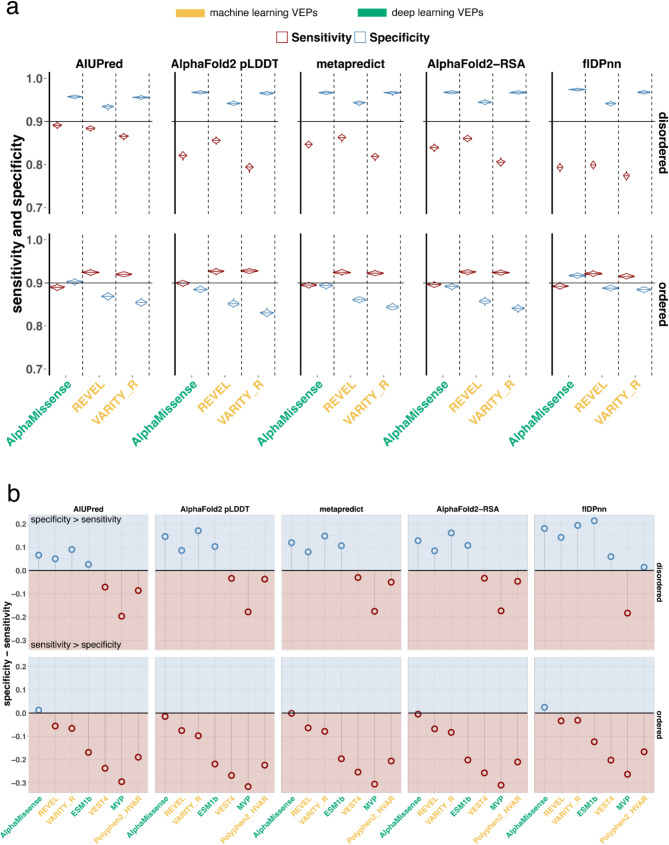
State-of-the-art VEPs tools have lower sensitivity but higher specificity for variants in disordered regions. **a**) Performance in terms of sensitivity and specificity (y-axis) of VEPs (x-axis) calculated on ClinVar variants according to disordered/ordereded class as predicted by AIUPred, AlphaFold2 pLDDT, metapredict, AlphaFold2-RSA and flDPnn. The violin plots show the performance of VEPs calculated on 200 bootstrap samples of 12530 variants each sampled with replacement and in equal proportion from the benign and pathogenic class and the disorder class (see Methods). The horizontal line in the violin shows the median value. To ease the comparison, a black horizontal line is set at 90% both for the sensitivity and specificity. The comparison with more VEPs is included in Supplementary Fig. 6 and in panel b. The best performing VEPs, namely AlphaMissense, VARITY, REVEL, are associated with higher specificity for variants in disordered regions and higher sensitivity in ordered regions. Interestingly, when disorder is measured with AIUPred the sensitivity of AlphaMissense does not vary between the disorder and the order groups. **b**) The difference between the median value of the distribution of specificity and sensitivity of panel a is plotted (y-axis). Values greater than 0 indicate that the specificity is higher than the sensitivity, and a value of 0.1 means that the specificity is 10% higher than the sensitivity. While values smaller than 0 indicate higher sensitivity. Traditional VEPs such as PolyPhen2 are still biased towards higher sensitivity, even for variants in disordered regions, whereas the performance of state-of-the-art VEPs, like AlphaMissense, is driven by specificity in disordered regions and sensitivity in ordered ones

Next, we investigated whether the performance of deep and machine learning VEPs such as AlphaMissense is consistent among variants associated with disordered residues. The stratification of VEPs' performance by disorder propensity highlighted that the top VEPs exhibit higher specificity than sensitivity when predicting variants in disordered regions (Fig. 6a). This observation is consistent across different metrics of disorder. Here, we considered top or best performing tools those with the smallest difference between sensitivity and specificity both in disordered and ordered regions, namely AlphaMissense, REVEL and VARITY (Fig. 6b). High sensitivity and high specificity are especially desirable in clinical diagnostics, where both false negatives and false positives rates should be minimized. The performance of VEPs by ROC-AUC and F1-score alone may lead us to conclude that variants in disordered regions are more accurately predicted, as both ROC-AUC and F1-score values are higher or as high as variants in ordered regions (Supplementary Fig. S7). This result would mask the actual unbalanced performance by class. In fact, the gap between sensitivity and specificity is highest in disordered regions measured with flDPnn (Fig. 6b), reaching up to 20% difference. Likewise, disorder measured by AF2 pLDDT and AlphaFold2-RSA results in more than 10% increase of specificity over sensitivity.

Strikingly, the discrepancy of AlphaMissense in the AF2 pLDDT (0,50] confidence group reaches almost 20% (Supplementary Fig. S5). A clear pattern emerges: variants in disordered regions are associated with higher specificity, and mutations in ordered regions with higher sensitivity. This pattern holds true for the latest deep and machine learning models such as AlphaMissense and REVEL, while VEST4 and PolyPhen2 remain biased towards pathogenic mutations also in the disordered set (Fig. 6b and Supplementary Fig. S6).

The already known bias of VEPs to over-predict pathogenic mutations [17, 25] seems to be complemented with the reversed bias of over-predicting benign mutations in disordered regions.

### State-of-the-art VEPs misclassify pathogenic mutations in IDRs

The higher specificity of VEPs for variants in disordered regions corresponds to lower sensitivity for variants in disordered regions (Fig. 6). AlphaMissense demonstrates the lowest sensitivity in predicting variants within C-terminal IDRs, a finding consistent across various definitions of IDRs (Supplementary Fig. S8). REVEL and VARITY achieve the lowest sensitivity for variants in the C-terminal IDR according to metapredict and in the C-terminal and N-terminal when IDRs are identified with AIUPred. The present observations do not reveal any consistent patterns or trends regarding the performance of VEPs across IDR groups.

As an example, we investigated a variant at the N-terminus of the heat-shock beta-1 protein encoded by the *HSPB1* gene. The N-terminus harbors known germline missense mutations that are associated with Charcot-Marie-Tooth disease (CMT) and Hereditary Motor Neuropathy (HMN) type II [58]. An example of a pathogenic variant is the mutation P39L (variant id: NM_001540.5(HSPB1):c.116 C > T (p.Pro39Leu)) which increases the propensity of helix formation and/or local contacts between the N-terminal regions [59]. Further, it prevents the dissociation of large oligomers of HSPB1 by phosphorylation [60]. While both ClinVar data and significant experimental evidence support the pathogenicity of this variant, the most recent and widely used VEPs, namely AlphaMissense, REVEL and VARITY, interpret this variant as benign, with a probability of pathogenicity of 0.29, 0.20 and 0.38 respectively. Although this is only one example, we detect across the whole dataset a decreased sensitivity for mutations in disordered regions compared to ordered regions (Fig. 6), thus indicating a higher false negative rate.

We further investigated the 3D model and secondary structure of the heat-shock beta-1 protein both as monomer and multimer, whose quaternary structure has been already extensively investigated [61–63]. Specifically, the work of Jehle et al. [64] led to a model of the N-terminal domain structure that consists of two alpha-helices and two beta strands that might not exist simultaneously and especially not in all oligomers.

**Fig. 7:**
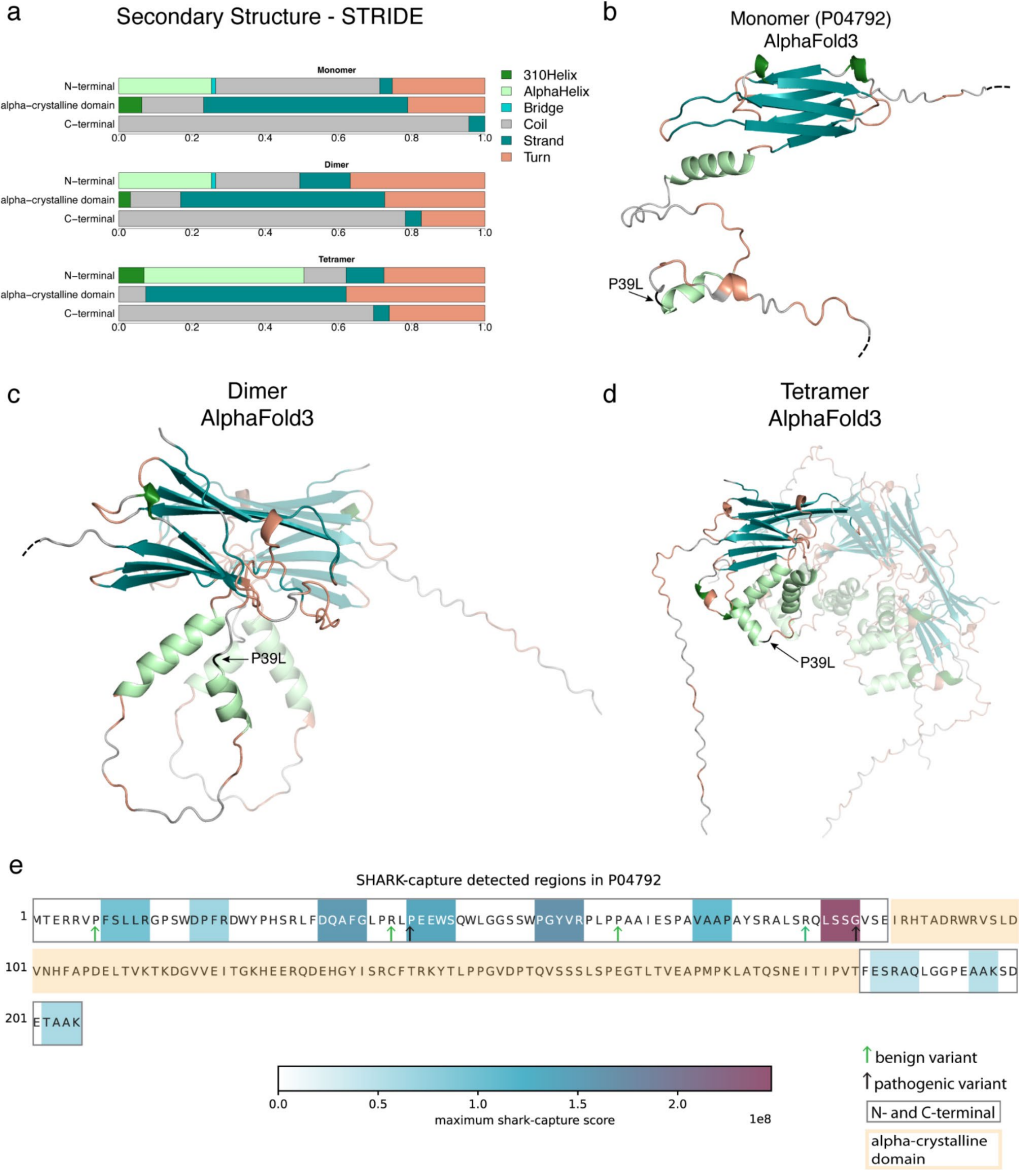
Disorder-to-order transitions predicted by AlphaFold3 may aid structure-based variant interpretation. **a**) The secondary structure annotation according to STRIDE for the AlphaFold3 3D model of the heat-shock beta-1 protein (HSPB1, Uniprot ID: P04792) in its monomeric (top), dimeric (middle) and tetrameric form (bottom). The disorder-to-order transition of the N-terminus is supported by the decrease of predicted coil elements in the dimeric and tetrameric organization of the protein with respect to its monomeric form and the corresponding increase of alpha-helices. In panels **b**), **c**) and **d**) the AlphaFold3 models of P04792 monomer, dimer and tetramer are shown respectively. Colors correspond to the legend in a) and only one chain is highlighted for the dimer and tetramer. The C-terminus is not displayed in its entirety as indicated by the dashed black lines of panel **b**) and **c**). The position with the variant P39L is highlighted in black. The disorder-to-order transition as predicted by STRIDE corresponds to the new alpha-helices of the dimer and tetramer visible in panel **c**) and **d**). **e**) Motif prediction of HSPB1 (P04792) using SHARK-capture. Conserved motifs of the disordered regions are colored by their SHARK-capture score, benign and pathogenic variants are marked. Pathogenic mutations fall into SHARK-capture motifs in the N- and C-terminal regions while benign mutations do not. The P39L pathogenic variant is located within a conserved PEEWS motif

The secondary structure assignment of STRIDE [65] describes the N-terminus as containing mostly disordered/coiled regions and turns, namely structural elements of 3 to 4 residues that connect helices and beta strands (Fig. 7a). However, the dimeric (Fig. 7c) and tetrameric (Fig. 7d) AlphaFold3 [66] 3D models of the heat-shock protein showed an increase in secondary structure elements compared to the monomeric structure, confirmed by STRIDE, and underpinning the experimental results (Fig. 7). In particular, in the dimeric form we observed a longer alpha-helix than in the monomer, and in the tetramer, we noticed another helix not present in the dimer and monomer (Fig. 7c). Thus, oligomerization promotes disorder-to-order transitions, specifically the formation of helices. Note that the 3D structural models of the C-terminal part of the protein remained unaltered across the different oligomeric conformations (Fig. 7a). Thus, the mutations at residue position 39 at the tip of the helix that elongates upon oligomerization may be correctly classified if the oligomeric structural state were considered. The above structural investigation highlights that oligomeric forms of the protein contain supplemental and perhaps relevant information to aid the discrimination of variants effect in disordered regions of the protein.

To further characterize the disordered region of HSPB1, we ran SHARK-capture, an alignment-free motif detecting tool specifically designed to identify conserved motifs in disordered regions. Focusing on the disordered parts of the sequence, namely the N- and C- terminal regions, we annotated the ten highest scoring motifs and found that two pathogenic variants, including P39L, fall into these motifs. In comparison, none of the benign mutations are located in a conserved region (Fig. 7e).

While this represents only one example, it serves as a proof of concept and highlights an alternative approach to study and characterize mutations in disordered regions.

## Discussion

Machine and deep learning VEPs are extensively and routinely benchmarked on well-characterized clinical variants to demonstrate their utility for clinical genetic diagnosis. The newest deep-learning VEP, AlphaMissense, predicts the pathogenicity of genetic variants with a remarkable 90% sensitivity and specificity, yet it was reported that the pathogenicity of genetic variants in disordered regions is poorly predicted [28, 29]. Here, we investigated the performance of AlphaMissense as well as other state-of-the-art VEPs on ClinVar mutations in disordered regions according to five different metrics of disorder, namely AIUPred [35], AlphaFold2 pLDDT [16], metapredict [36, 37], AlphaFold2-RSA [38] and flDPnn [39].

Our work confirmed that pathogenic mutations in disordered regions are prevalent [5, 6]: we found that more than 10% of disease mutations are associated with disordered residues, except for flDPnn (Fig. 3). Interestingly, we observed that the first N-Methionine site, which is often predicted as disordered, is the residue with the most annotated variants, and that more than 90% of mutations at this site are pathogenic (Fig. 5a and Supplementary Fig. S3). We found that the interpretation of variants effect at this site is poor across all VEPs (Fig. 5c) but it can be improved by considering the distance to the second Methionine in the protein sequence (Fig. 5b), as reported [56]. While it remains unclear why mutations at the N-Methionine site are so prevalent, we concur with previous studies that the assessment of pathogenicity at this site should be handled independently from mutations at any other site [57].

After excluding mutations at the N-Methionine site, we assessed the performance of VEPs for variants located in disordered regions. Our analysis revealed that the performance in disordered regions is more unbalanced compared to that in ordered regions, with higher specificity than sensitivity (Fig. 6). The gap between specificity and sensitivity remains large across disorder metrics; when disorder is measured with AIUPred, the sensitivity in disordered and ordered regions are not different (Fig. 6a). On the other hand, when disorder is measured with AlphaFold2 pLDDT, AlphaFold2-RSA, metapredict and flDPnn the difference between sensitivity and specificity reaches at least 10% (Fig. 6b). While this observation underscores the lack of a clear and standardized definition of disorder, all five disorder computational tools reveal a more pronounced imbalance of VEPs' performance in disordered regions.

The higher sensitivity in ordered regions corresponding to higher specificity in disordered ones suggests that AlphaMissense, like other state-of-the art VEPs, relies on the traditional conservation paradigm to interpret variant effects, where mutations in conserved domains tend to be predicted as pathogenic, whereas those in less conserved, alignable and structured regions tend to be predicted as benign. This holds true both for supervised models such as REVEL, whose features consist of pathogenicity predictions as well as conservation scores, and for unsupervised deep learning models that learn the probability of protein sequences from the protein’s specific evolutionary history represented by its multiple sequence alignment (MSA). Alignment-free models such as ESM1b that train on all available protein sequences without explicit requirement of homology are nonetheless learning evolutionary constraint of proteins [19, 67] and as such are biased towards high sensitivity in ordered regions and high specificity in disordered ones (Figs. 6 and 7). However, since the case of pathogenic mutations in disordered regions falls outside this current framework, such predictors tend to over-predict variants as benign, thereby increasing the false negative rate.

The current observations convey a significant message regarding the implications of VEPs in clinical decision-making. AlphaMissense achieves a high and balanced performance for variants in ordered and conserved regions, representing a substantial enhancement over VEPs that attain higher sensitivity at the expense of a high false positive rate (Figs. 6 and 7). However, given the performance bias observed in disordered regions, it is recommended that secondary structure information and disorder scores be reported. This would allow clinicians and researchers to further investigate the functional impact and disease relevance of variants in disordered regions using other computational tools and experiments.

The performance of VEPs is biased towards high specificity in disordered regions and may improve if features specifically designed for such regions are implemented. The case described in the results of the P39L mutation in the disordered N-terminal domain of the heat-shock beta-1 protein is an example (Fig. 7). The functional and structural importance of the N-terminus for the protein oligomerization is extensively documented and from our qualitative exploration, it seems that it might be beneficial to consider the structural conformation of the N-terminus in the dimeric and multimeric form (Fig. 7) which is now possible, but not yet scalable, with AlphaFold3 [66]. In the future, the design of features that capture residue functional importance in the tertiary and quaternary structure of a protein may further improve the accuracy of variant effect interpretation.

## Conclusion

Predicting pathogenic variants in disordered regions remains challenging because the mechanisms of pathogenicity in such regions have not yet yielded a paradigm similar to the one for mutations occurring in ordered regions. Especially given their reduced sequence conservation due to the lack of an evolutionary constraint to maintain structure. Accordingly, our study highlights the lack of features for VEPs aimed specifically for disordered regions, which manifests in lowered sensitivity and a discrepancy between sensitivity and specificity for most VEPs. Ultimately, given the criticalroles of IDRs in binding events such as signaling, regulation and the formation of protein complexes, development and inclusion of IDR-specific features that describe the 3D structure of the protein as well as its interactions in protein complexes are needed to improve variant effect assessment in IDRs.

## Electronic supplementary material

Below is the link to the electronic supplementary material.

Supplementary Material 1

## Data Availability

All code and data are available at the gitlab https://git.mpi-cbg.de/tothpetroczylab/idrs_veps.
