# Supplementary material for: Deep learning tools predict variants in disordered regions with lower sensitivity
Source: BMC Genomics. 2025 Apr 12;26:367. doi: 10.1186/s12864-025-11534-9 (PMC11992697; doi:10.1186/s12864-025-11534-9)
Supplement: Supplementary file 1 — Supplementary Material 1 [file 12864_2025_11534_MOESM1_ESM.pdf]

Supplementary Information

for

Deep learning tools predict variants in disordered regions with lower sensitivity

Federica Luppino[1,2], Swantje Lenz[1,2], Chi Fung Willis Chow[1,2,3], Agnes Toth-Petroczy[1,2,3]

1. Max Planck Institute of Molecular Cell Biology and Genetics, Pfotenhauerstrasse 108, 01307 Dresden, Germany
2. Center for Systems Biology Dresden, Pfotenhauerstrasse 108, 01307 Dresden, Germany
3. Cluster of Excellence Physics of Life, TU Dresden, 01062 Dresden, Germany

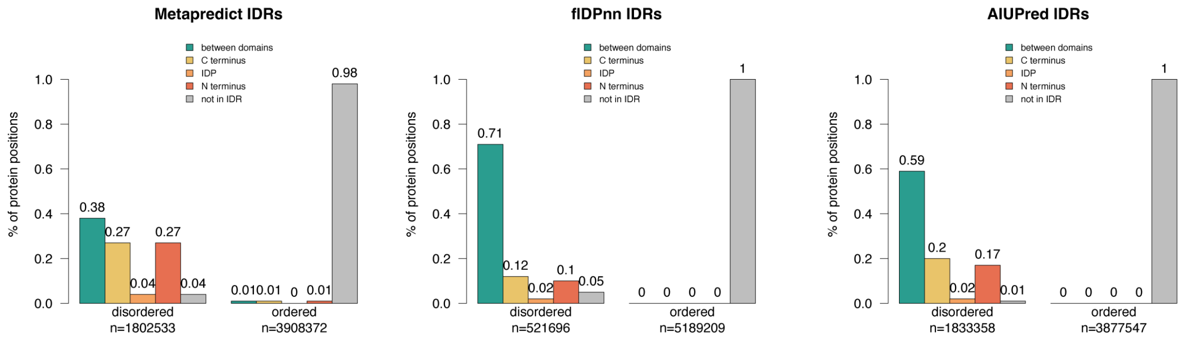

Supplementary Fig. 1. Region-level and residue-level disorder predictions are equivalent.

On the x-axis the residue-level disorder scores as a binary group, namely disordered and ordered for all 7466 proteins. On the y-axis the proportion of protein positions in the different protein groups: C-terminal, N-terminal, between domains Intrinsically Disordered Regions (IDRs), not in an IDR and Intrinsically Disordered Proteins (IDPs). The minimal length of the IDRs is 10. IDRs shorter than 10 residues are included in the 'not in IDR' category. Only 4%, 5% and 1% disordered residues are not contained in an IDR according to metapredict, flDPnn and AIUPred respectively.

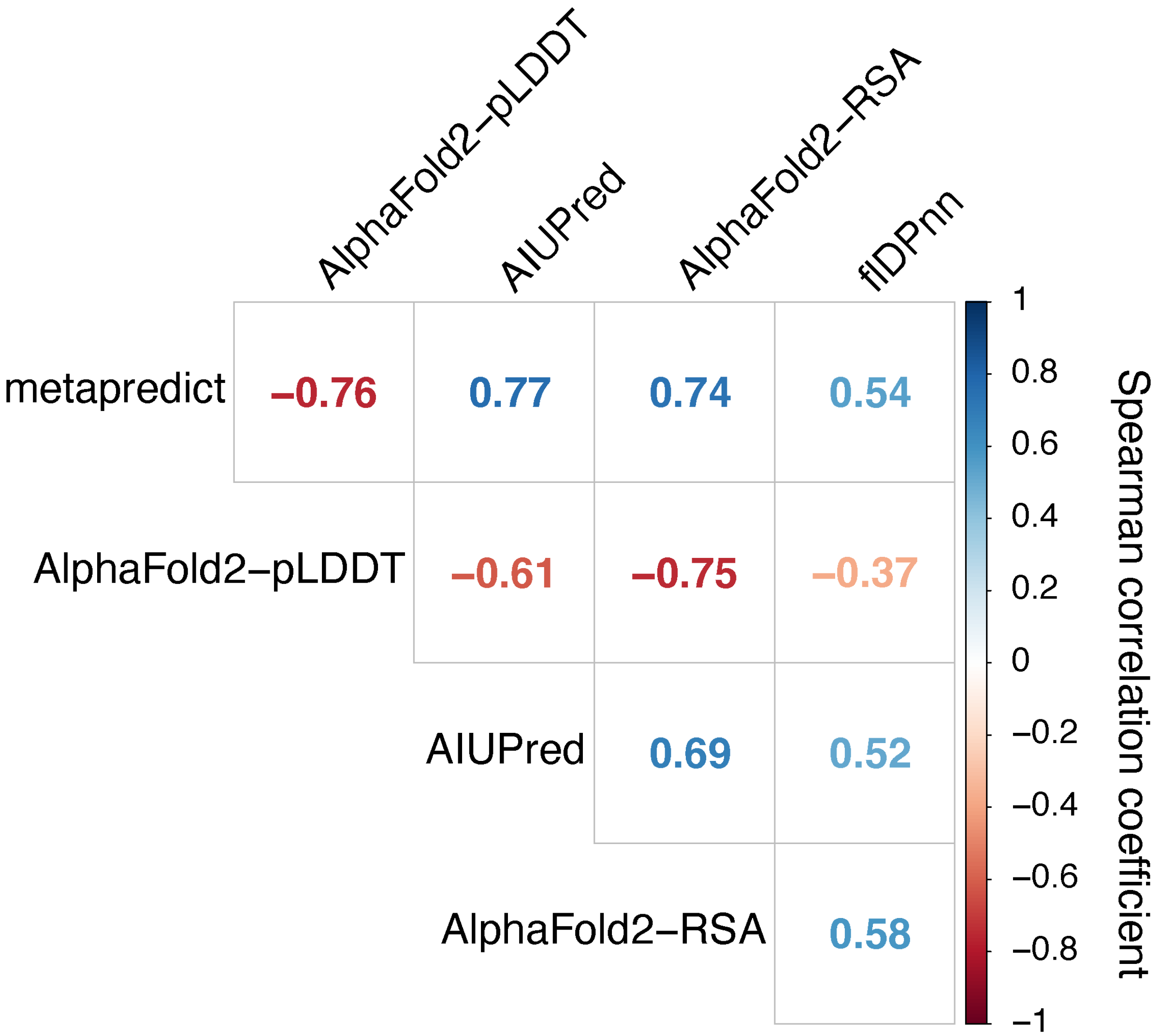

Supplementary Fig. 2. Correlation between computational disorder predictor scores for all residues in 7466 ClinVar proteins.

The Spearman correlation coefficients between the five different computational disorder predictors is shown with a correlation matrix. All the ten p-values of the correlation tests are significant at the Bonferroni corrected threshold of 0.01, namely 0.001 (0.01/10). Blue (red) values show positive (negative) correlation. The lowest correlation is between flDPnn and AlphaFold2-pLDDT while the highest is the one of metapredict and AIUPred.

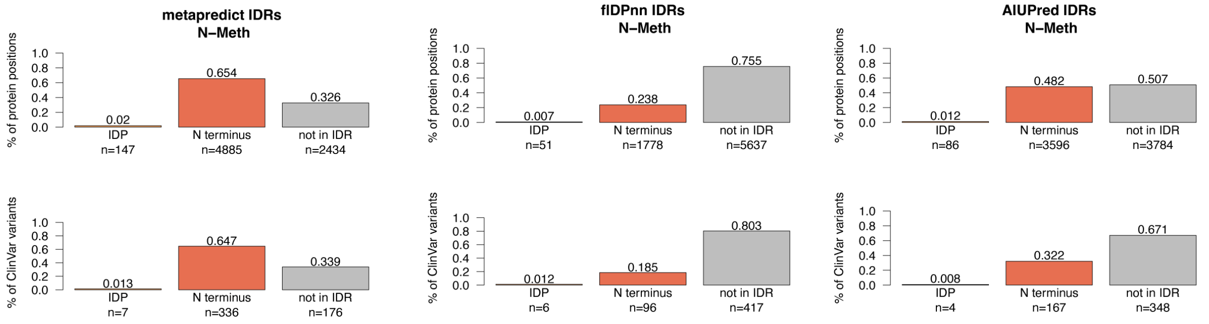

Supplementary Fig. 3. Variants phenotypic effect according to IDRs categories and N-Methionine IDRs.

On the x-axis the category for N-methionine sites, namely IDP, N-terminal IDR or not in an IDR. The first row is calculated for all N-methionine sites while the second row focuses on N-Methionine sites associated to ClinVar variants.

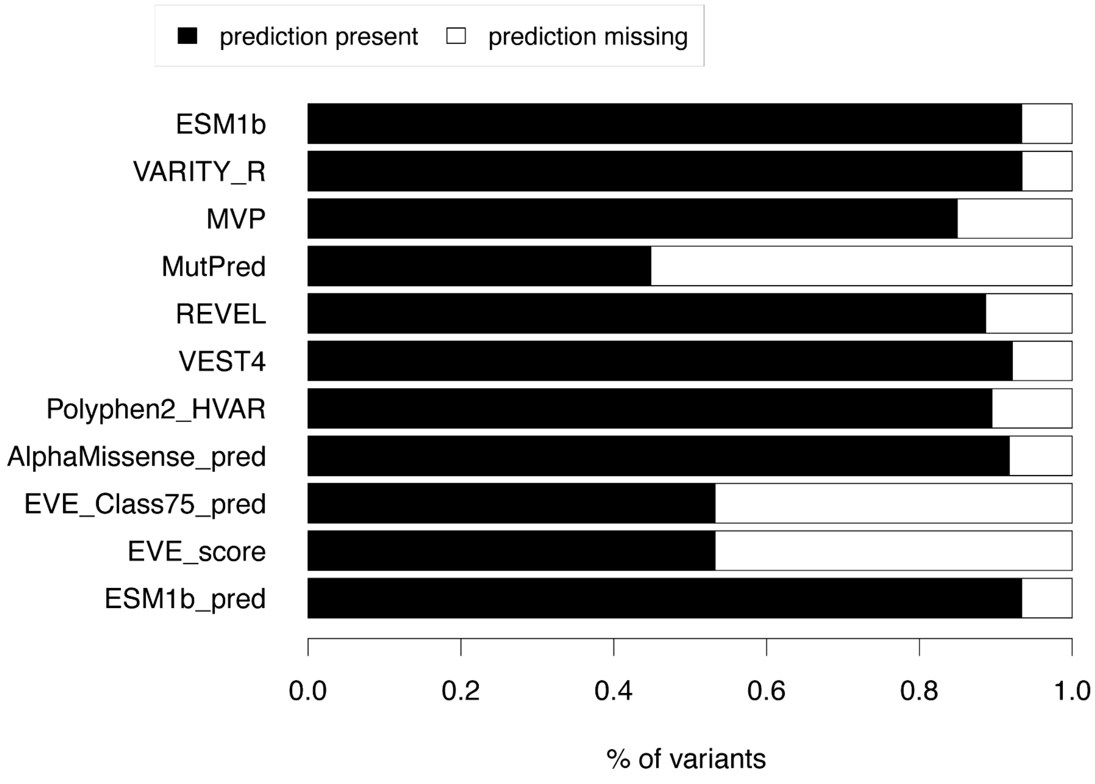

Supplementary Fig. 4. VEPs missing predictions for ClinVar variants.

Most VEPs have predictions for more than 90% of ClinVar variants except for EVE and MutPred that have prediction scores for around 50% of variants.

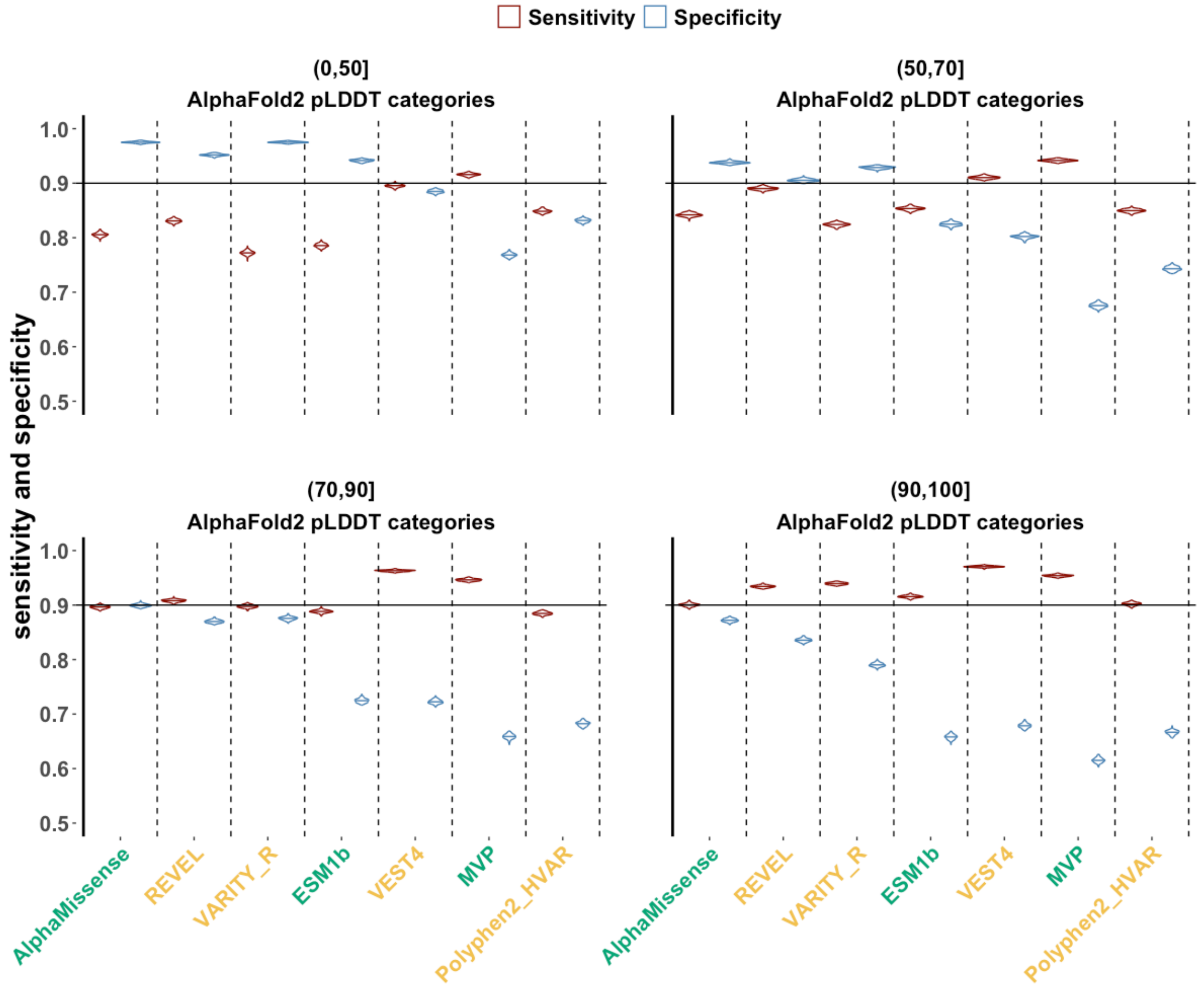

Supplementary Fig. 5. VEPs performance on ClinVar variants by AlphaFold2 pLDDT confidence categories.

The same representation of main Fig. 4 now stratified by AlphaFold2 pLDDT confidence categories. The discrepancy between sensitivity and specificity is highest in the category (0,50], except for VEST4. In the category (50,70] all tools maintain the disproportion between the two metrics except for REVEL. In the group (70,90] AlphaMissense, REVEL and VARITY present the most balanced performance that is kept only by AlphaMissense in the most confident category (90,100]. All the remaining tools show much higher sensitivity than specificity.

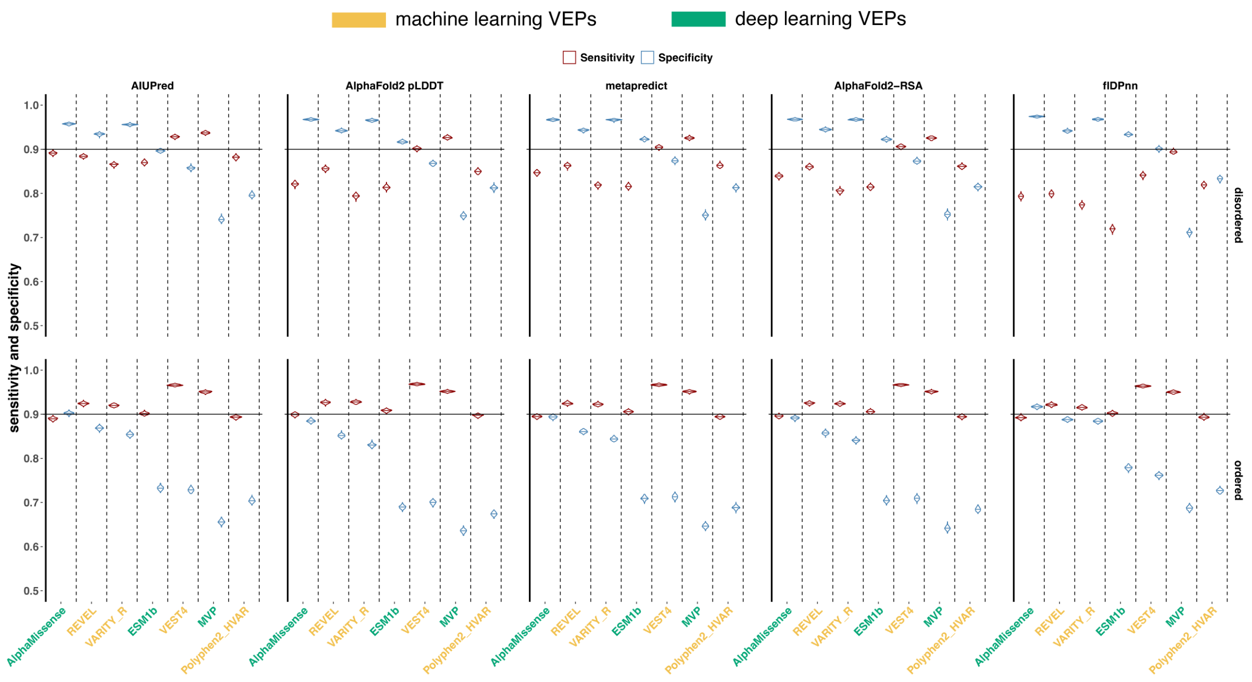

Supplementary Fig. 6. Traditional VEP tools are still biased towards higher sensitivity, also for variants in disordered regions.

Performance in terms of sensitivity and specificity (y-axis) of VEPs (x-axis) calculated on ClinVar variants according to disordered/ordereded class as predicted by AIUPred, AlphaFold2 pLDDT, metapredict, AlphaFold2-RSA and flDPnn. The violin plots show the performance of VEPs calculated on 200 bootstrap samples of 12530 variants each sampled with replacement and in equal proportion from the benign and pathogenic class and the disorder class (see Methods). The horizontal line in the violin shows the median value. To ease the comparison, a black horizontal line is set at 90% both for the sensitivity and specificity.

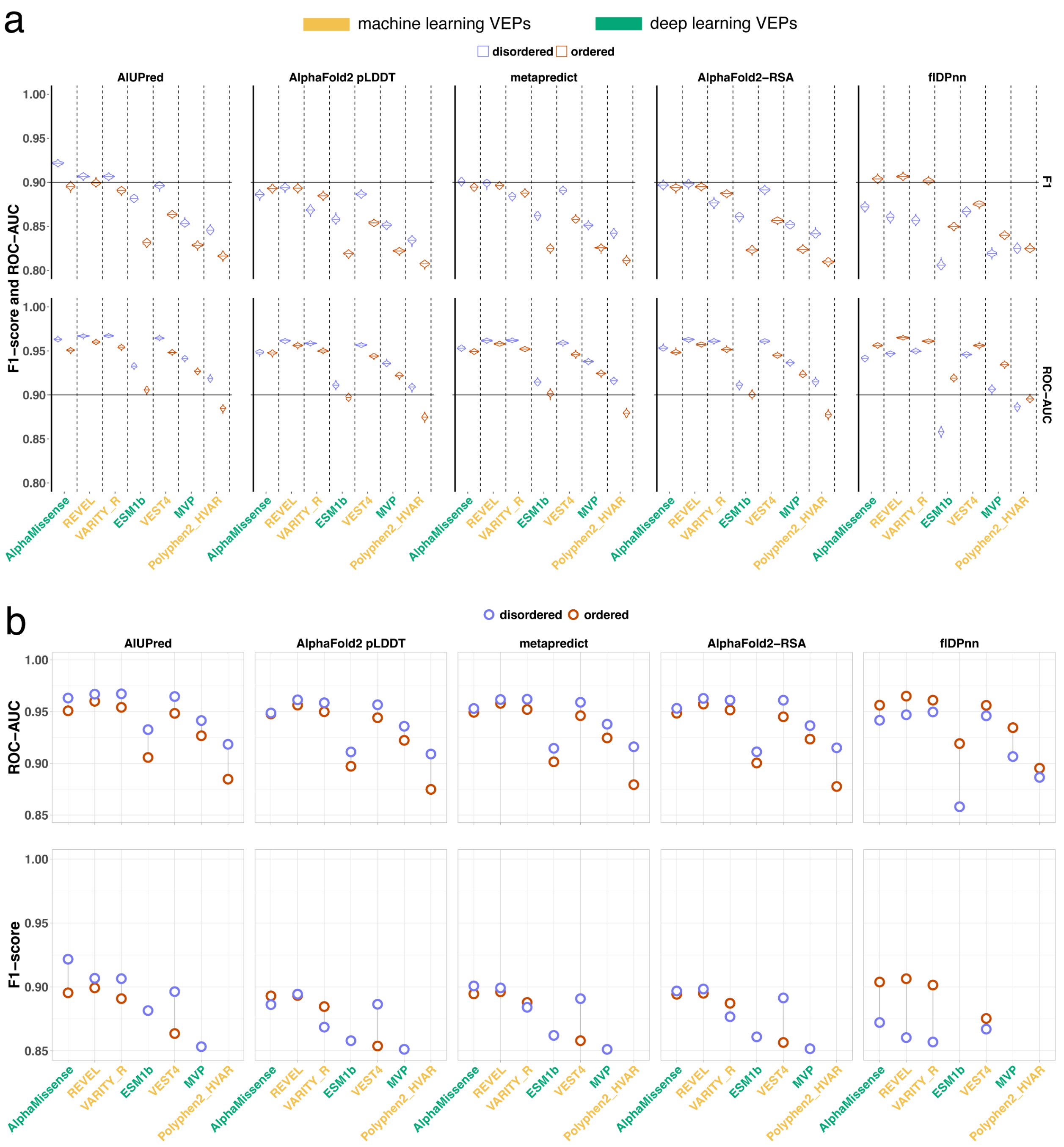

Supplementary Fig. 7. The discrepancy between F1-score and ROC-AUC for disordered regions is small.

a) Performance in terms of F1-score and ROC-AUC (y-axis) of VEPs (x-axis) calculated on ClinVar variants according to disordered/ordereded class as predicted by AIUPred, AlphaFold2 pLDDT, metapredict, AlphaFold2-RSA and flDPnn. The violin plots show the performance of VEPs calculated on 200 bootstrap samples of 12530 variants each sampled with replacement and in equal proportion from the benign and pathogenic class and the disorder class (see Methods).

The horizontal line in the violin shows the median value. To ease the comparison, a black horizontal line is set at 90% both for the sensitivity and specificity.
b) The median values of the distribution of F1-score and ROC-AUC of panel a is plotted (y-axis).

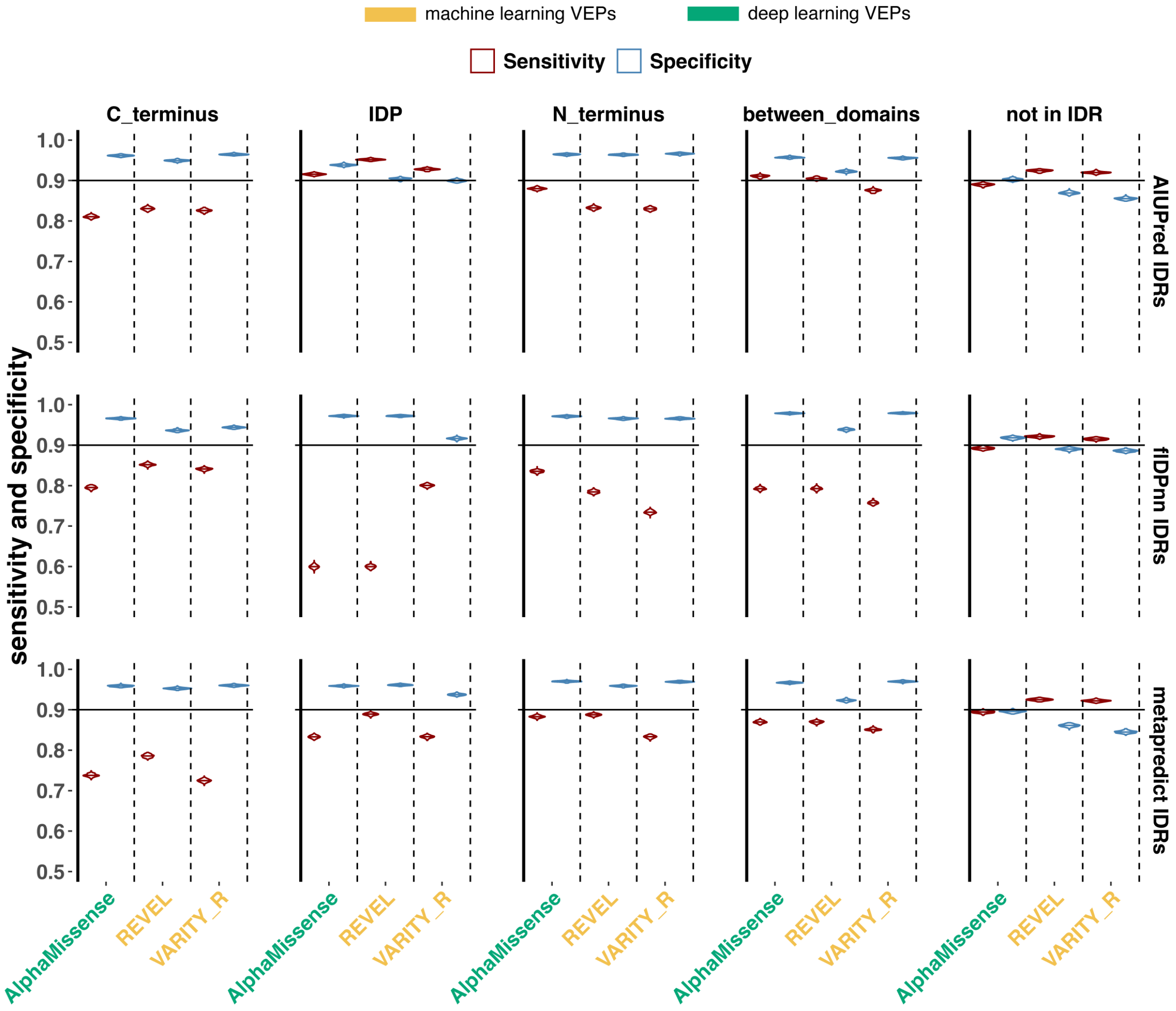

Supplementary Fig. 8. VEPs performance for variants in IDR groups depends on the disorder tool.

Performance in terms of sensitivity and specificity (y-axis) of VEPs (x-axis) calculated on ClinVar variants according to IDRs groups as predicted by AIUPred, metapredict, and flDPnn (see Methods for further details on IDRs definition). The violin plots show the performance of VEPs calculated

on 200 bootstrap samples of 12530 variants each sampled with replacement and in equal proportion from the benign and pathogenic class and the disorder class (see Methods). The horizontal line in the violin shows the median value. To ease the comparison, a black horizontal line is set at 90% both for the sensitivity and specificity.

Supplementary References

1. Jumper J, Evans R, Pritzel A, Green T, Figurnov M, On ne be rg er OR, et al. Highly accurate protein structure prediction with AlphaFold. Nature. 2021. https://doi.org/10.1038/s41586-021-03819-2.
